# Auditory Cue Effects on Gait-Phase-Dependent Electroencephalogram (EEG) Modulations during Overground and Treadmill Walking

**DOI:** 10.3390/s24051548

**Published:** 2024-02-28

**Authors:** Kittichai Tharawadeepimuk, Weerawat Limroongreungrat, Metaneeya Pilanthananond, Ampika Nanbancha

**Affiliations:** 1 College of Sports Science and Technology, Mahidol University, Nakhon Pathom 73170, Thailand; kittichai.tha@mahidol.edu (K.T.); weerawat.lim@mahidol.edu (W.L.); 2 Department of Physiology, Faculty of Science, Mahidol University, Bangkok 10400, Thailand; metaneeya.pil@mahidol.edu

**Keywords:** electroencephalogram (EEG), gait cycle, walking, ambulation

## Abstract

Walking rehabilitation following injury or disease involves voluntary gait modification, yet the specific brain signals underlying this process remains unclear. This aim of this study was to investigate the impact of an auditory cue on changes in brain activity when walking overground (O) and on a treadmill (T) using an electroencephalogram (EEG) with a 32-electrode montage. Employing a between-group repeated-measures design, 24 participants (age: 25.7 ± 3.8 years) were randomly allocated to either an O (*n* = 12) or T (*n* = 12) group to complete two walking conditions (self-selected speed control (sSC) and speed control (SC)). The differences in brain activities during the gait cycle were investigated using statistical non-parametric mapping (SnPM). The addition of an auditory cue did not modify cortical activity in any brain area during the gait cycle when walking overground (all *p* > 0.05). However, significant differences in EEG activity were observed in the delta frequency band (0.5–4 Hz) within the sSC condition between the O and T groups. These differences occurred at the central frontal (loading phase) and frontocentral (mid stance phase) brain areas (*p* < 0.05). Our data suggest auditory cueing has little impact on modifying cortical activity during overground walking. This may have practical implications in neuroprosthesis development for walking rehabilitation, sports performance optimization, and overall human quality-of-life improvement.

## 1. Introduction

Ambulation or walking is a fundamental motor behavior requiring intricate coordination between skeletal muscles and neural pathways across the brain [1,2,3]. While the subcortical brain traditionally governs basic locomotion, recent studies highlight the significant role of the cerebral cortex in this process [4,5,6]. Patients with conditions impairing walking abilities, such as stroke, Parkinson’s disease, and Huntington’s disease, often benefit from rehabilitation techniques aimed at improving ambulation [6]. Auditory cues have emerged as a promising tool in walking rehabilitation, offering various benefits including improved physical fitness, posture, stress reduction, and navigation [7,8]. These cues can range from exercise technique instruction, motivational prompts, entertainment features, and safety alerts to guide walking behavior. Notably, auditory cueing has been linked to enhance cortical activity during walking recovery, with increased engagement observed across various cortical regions [9,10].

Previous research has demonstrated that rhythmic auditory cueing leads to increased cortical activity during walking recovery [11]. In a study involving stroke patients, the integration of visual and sensorimotor information across various cortical regions was observed [10]. Researchers used functional near-infrared spectroscopy (fNIRS) to measure changes in oxygenated hemoglobin concentrations in different brain regions. However, due to fNIRS’s limited spatial resolution and challenges in signal interpretation, it is unsuitable for examining changes in cortical activation across different phases of the gait cycle. In contrast, electroencephalography (EEG) directly records neuronal electrical activity, offering superior spatial resolution. Moreover, EEG’s millisecond-level temporal resolution permits detailed information about the timing of neuronal events [12]. Recent EEG research has examined the fundamental aspects of brain activity during various ambulatory conditions [13]. For example, in stroke survivors undergoing robot-assisted gait therapy, the EEG delta frequency band (0.5–4 Hz) has been associated with sensorimotor information, deep sleep states, and the execution of movement [14,15,16,17,18]. While cortical modulations are known to be linked to the gait cycle phase, individual components such as the swing and stance phases are not commonly investigated [19,20,21,22].

In rehabilitation settings, individuals may undergo ambulation either overground or on a treadmill, adjusting their pace based on self-selection or following predetermined cues like a metronome. Currently, only one study [23] has compared EEG activity between these two surfaces during the gait cycle, finding increased cortical activity in the theta frequency band with self-selected speeds on both surfaces. However, it remains unclear whether overground walking with rhymical auditory cues, serving as an anticipatory and external frame reference [24], results in different EEG brain activity compared to treadmill walking at a constant speed with minimal engagement [25]. The potential variations in sensorimotor-related information, motor intention, and cognitive demands may influence cortical activity across different walking conditions. Therefore, investigating EEG amplitude modulation of sensorimotor processing during varied walking tasks could offer novel rehabilitation interventions to effectively modulate gait-related brain activity. Integrated EEG analysis from gait studies may contribute methodologically to establishing new standards for studying the neural correlates of locomotion.

Accordingly, we aimed to harness the technological advances offered by EEG amplitude analysis to investigate brain activity modulation during overground (O) and treadmill (T) walking across the gait cycle. We specifically investigated two speed control conditions: self-selected speed control (sSC) and speed control (SC), representing walking without and with an auditory guidance, respectively. Furthermore, instead of pooling electrodes, we utilized EEG-normalized amplitude for individual electrodes to improve spatial resolution [26], enabling a more detailed analysis of brain activity during walking [17]. We hypothesized that distinct differences would emerge within and between groups in the EEG delta frequency band, reflecting the impact of auditory cued walking versus normal walking.

## 2. Materials and Methods

### 2.1. Participants

Twenty-four healthy volunteers (12 males and 12 females; age: 25.7 ± 3.8 years; height: 166.7 ± 7.5 cm; body mass: 64.6 ± 14.2 kg) participated in this study. All participants were right-leg and right-arm dominant. Participant inclusion criteria were: (1) normal joint alignment and gait pattern, (2) Q-angle within 10°–20°, and (3) leg length discrepancy not exceeding 2 cm [27]. Exclusion criteria were (1) knee joint deformity, (2) feet supination or overpronation, (3) pelvic side shift, (4) recurrent or recent injuries of the lower extremities, (5) musculoskeletal problem on either leg within the past three months, and (6) history of neurological disease or vestibular/visual disturbance. The mini-mental state examination (MMSE) and the participant’s medical history were evaluated prior to recruitment. All participants provided written informed consent prior to their participation in this study. The experiments were approved and performed in accordance with the research ethics committee of Mahidol University—Central Institutional Review Board (COA MU-CIRB 2022/060.2005).

This study used a pseudonym for recording the wearable sensor data, thereby anonymizing the identity of the participants. In accordance with informed consent, the data were collected for non-commercial research purposes. The dataset is available at https://osf.io/2R9BA/, accessed on 9 January 2024. Any use or sharing of the data, as well as any derived use, must strictly adhere to the same usage guidelines (for non-commercial research purposes, without attempts at identification, and with limited disclosure). Violations of these usage regulations will be subject to legal consequences for the individual users, as the dataset publisher bears no responsibility.

### 2.2. Experimental Setup and Design

Employing a between-subject repeated-measures design, 24 participants were randomly allocated into either an O (*n* = 12, 6 males, 6 females) or T (*n* = 12, 6 males, 6 females) walking group. The participants in both groups were asked to perform the same two walking conditions (sSC, SC). The sSC condition acted as the control condition within each group (i.e., non-auditory cue), and required participants not to exceed a walking speed of 0.8 m/s (2.88 km/h), as previous research has indicated movement artifacts become more pronounced beyond this threshold [28]. In the SC condition, the participants were asked to walk in time with a metronome (auditory cue) set at 0.55 m/s (1.98 km/h). The participants in the O group were instructed to walk directly forwards 100 m and not to turn around. Each walking sequence corresponded to walking for a duration no greater than 3 min. This approach can minimize EEG artifacts during walking [17,28,29,30]. The test instruments, tasks, and experimental procedures were explained and demonstrated to the participants prior to data collection.

### 2.3. Data Collection

**Figure 1:**
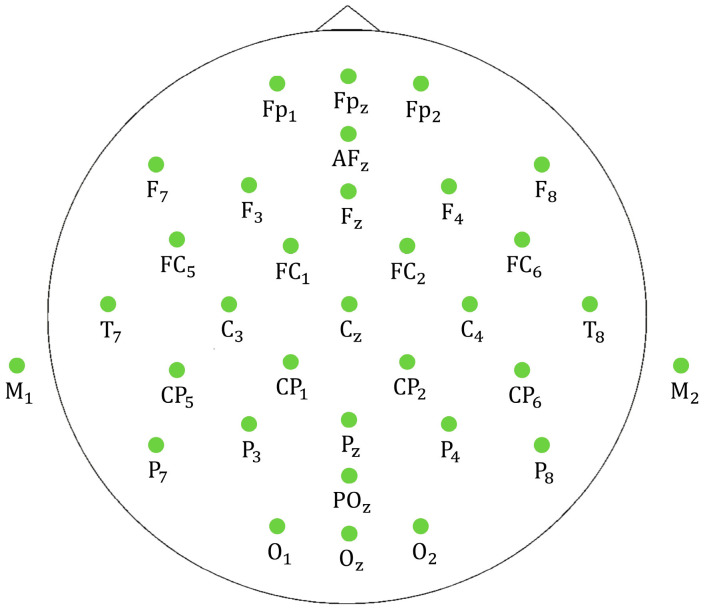
Montage of the EEG system in the experiment, 32 electrodes.

EEG signals were recorded during walking using a portable EEG system (eego™mylab; ANT Neuro) with a 32-electrode EEG cap. The EEG electrode positions included Fp_1_, Fp_2_, Fp_z_, F_3_, F_4_, F_7_, F_8_, F_z_, FC_1_, FC_2_, FC_5_, FC_6_, C_3_, C_4_, C_z_, T_7_, T_8_, CP_1_, CP_2_, CP_5_, CP_6_, P_3_, P_4_, P_7_, P_8_, P_z_, PO_z_, O_1_, O_2_, O_z_, M_1_, and M_2_, along with the reference electrode (REF = CP_z_) and the ground electrode (AF_z_), as shown in Figure 1. The electrode impedances were maintained below 20 kΩ, as recommended [28]. The EEG data were sampled at 512 Hz. In addition, inertial measurement unit (IMU) signals were recorded using a wireless IMU system (Noraxon; Ultium Motion) with 7 sensors placed on the sacrum, L|R thigh, L|R shank, and L|R second metatarsal (MyoMotion ISB-compliant modeling). The IMU data were sampled at 200 Hz, and smoothed via a Kalman filter approach [31]. EEG and IMU data of each participant were recorded for 3 min together in the sSC and SC conditions.

### 2.4. EEG Analysis

All data were analyzed using custom-written routines programs in MATLAB (v2020b, MathWorks, Natick, MA, USA). The raw EEGs were bandpass-filtered (within the range of 0.5–100 Hz for delta band, 4.5–100 Hz for theta band, 8–100 Hz for alpha band, and 13–100 Hz for beta band, using a fourth-order Butterworth filter), and notch-filtered (at 50 Hz, using a fourth-order Butterworth filter). These two filters were used for preparation of EEG data before using ICA algorithms, which greatly improved the quality of artifact separation [32,33]. Next, to identify brain and non-brain sources mixing in the EEGs, the preprocessed EEG data were decomposed using ICA using a “fastICA” algorithm. The procedural sequence in the fastICA algorithm encompassed the subsequent steps: centering (remean), decorrelation (covariance matrix), principal component analysis: PCA (pcamat), normalization (whitening), and ICA (fpica). Using the fastICA algorithm, five ICs were estimated. This process can remove the artifact ICs, which included two ICs originating from ocular, one IC from glossokinetic, and two ICs from muscular activity during walking-related movement [33,34]. Then, the EEG data were low-pass-filtered (using 4 Hz cutoff for delta band, 7.5 Hz cutoff for theta band, 12 Hz cutoff for alpha band, and 30 Hz cutoff for beta band, using a fourth-order Butterworth filter).

**Figure 2:**
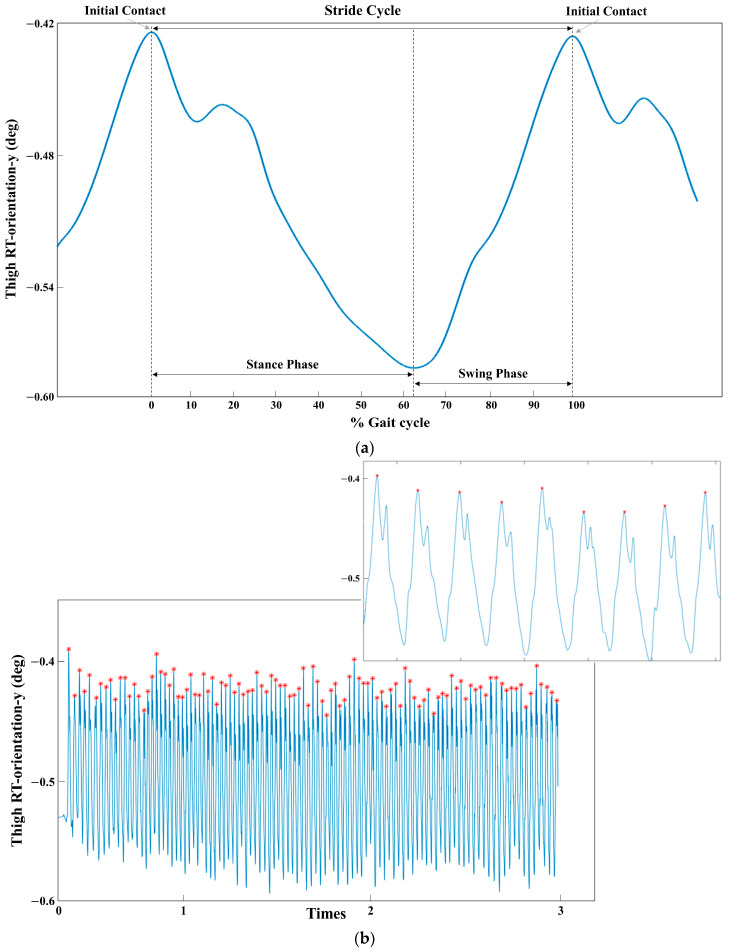
(**a**) The average of 105 gait cycles from a participant walking in the TsSC condition over 3 min; (**b**) a typical example of raw IMU data from a participant walking in the TsSC condition. The red mark indicates the peak of the IMU data, representing the initial contact point of the gait cycle.

To analyze EEG signals within each gait cycle, IMU data were used to identify initial contact, defined as the maximal right thigh angle (Noraxon MyoMotion-Segments Thigh RT-orientation-y (Figure 2). EEG and IMU signals were collected simultaneously and synchronized with digital markers. In the IMU signal, each gait cycle was defined as the time elapsed between two subsequent initial contact points and divided into the stance phase and swing phase using the minimum right thigh angle. The duration of each subphase was then expressed as a percentage of the gait cycle [35,36]. EEG data corresponding to each gait cycle were subsequently selected from the locations corresponding to the initial phase. All within-stride EEG data were then amplitude-normalized to the local peak value and time-normalized to 100 data points. Additionally, the data were ensemble-averaged across gait cycles for each participant. The processed EEG data yielded a matrix of 30 rows, corresponding to the 30 active EEG electrodes, and 100 columns corresponding to the 100 time points (percentage of gait cycle).

Furthermore, the EEG data of each active electrode were ensemble-averaged across participants for each group and walking condition (TsSC: treadmill walking with self-selected speed control, TSC: treadmill walking with speed control, OsSC: overground walking with self-selected speed control, and OSC: overground walking with speed control) to represent typical traces at each location. These data were used to display the direction and magnitude of the changes in gait-phase-related brain activities, which are represented in a colormap using an arbitrary unit (a.u.) scale. The average and standard deviation (SD) of amplitudes of the EEG data were used to calculate amplitude differences among groups and walking conditions.

### 2.5. Statistical Analysis

All statistical tests were performed in MATLAB. To assess the normality of the data, Shapiro–Wilk tests were initially applied, revealing an abnormal distribution of the data. Subsequently, statistical non-parametric mapping (SnPM) analysis was employed for all time-series EEG electrode data. We were primarily interested in comparing group x condition interaction effects, specifically the within-group interaction effects (OSC vs. OsSC and TSC vs. TsSC) to compare walking with and without an auditory cue on EEG brain activity across the gait cycle. Moreover, we were interested in comparing the between-group effects (OSC vs. TSC) to examine the impact of the walking surface on EEG brain activity when employing the auditory cue. Therefore, a two-way between-subject (group: O, T) analysis of variance (ANOVA) with repeated measures (condition: sSC and SC) was conducted to explore differences in EEG amplitude within each frequency band as part of the SnPM analysis. Where significant main interaction effects were identified, a post hoc test with a Bonferroni correction was applied. Only the results demonstrating statistical significance or important interactions are presented in this study. All data are reported as means ± standard deviation (SD) unless otherwise stated. The significance level was set at *p* < 0.05.

## 3. Results

**Figure 3:**
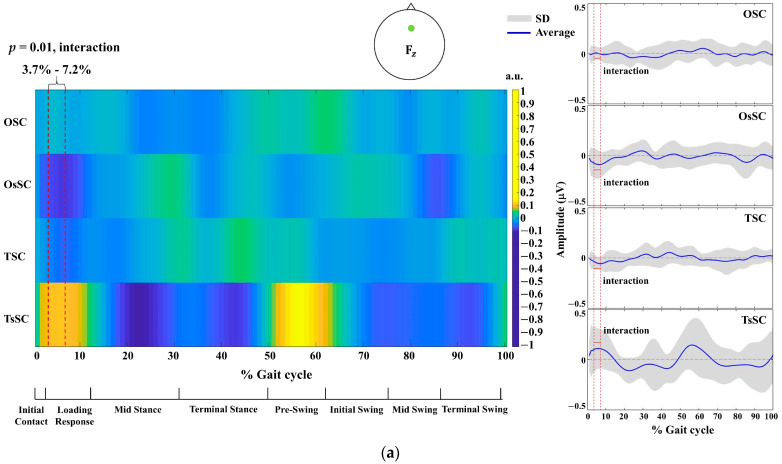

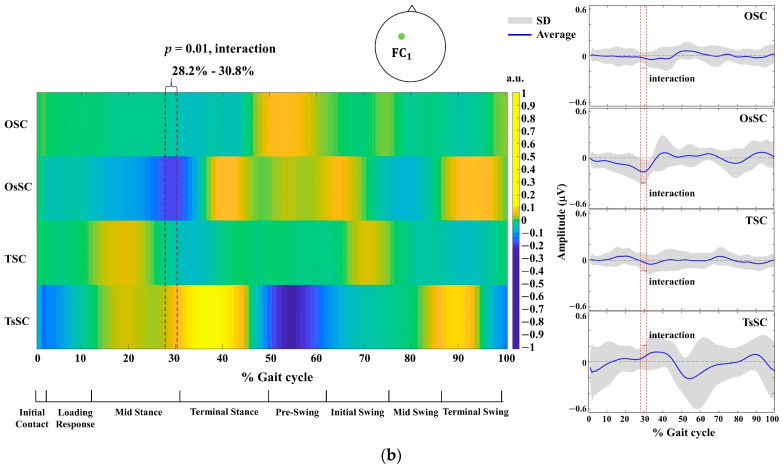
A colormap showing the average (blue line), and SD (gray shade) brain activity of the participants in each walking task: TsSC, TSC, OsSC, OSC. The colormap represents gait-phase-related brain activity changes in arbitrary units (a.u.) in the AmEEGDel (0.5–4 Hz). The average and SD represent gait-phase-related brain activity changes in microvolts (μV). Brain activities across the phases of the gait cycle showing EEG magnitude and direction at (**a**) F_z_ and (**b**) FC_1_ positions. The red vertical line demonstrates the percentage phase of the gait cycle where a significant interaction effect between groups (O, T) and conditions (sSC, SC) was identified.

SnPM analysis revealed differences in EEG time series at several brain locations in the amplitude in the EEG delta frequency band (AmEEGDel; 0.5–4 Hz). A significant interaction effect was found in EEG time-series data at the F_z_ position (*p* = 0.01, F* = 9.953), with a significant increase in EEG magnitude between TsSC and OsSC (*p* = 0.039; Figure 3a) at 3.7% to 7.2% of the gait cycle, corresponding to the loading response phase. A significant interaction was also found at the FC_1_ position (*p* = 0.01, F* = 9.135), with a significant increase in EEG magnitude between TsSC and OsSC (*p* = 0.039; Figure 3b) at 28.2% to 30.8% of the gait cycle, corresponding to the mid stance phase. However, no within-group differences in EEG time-series activity were found across brain locations between OSC and OsSC (all *p* > 0.05) or TSC and TsSC (all *p* > 0.05) conditions during the gait cycle. Similarly, there was no significant between-group difference in EEG time-series activity observed across brain regions in the OSC and TSC conditions across the gait cycle (all *p* > 0.05). The number of steps performed over the 3 min walking task within each group and condition are displayed in Table 1.

## 4. Discussion

In this study, we examined how an auditory cue influences brain activity during overground and treadmill walking, using EEG time-series data. Our main findings are as follows: (1) no significant differences were observed across any brain region between the O and T groups in the SC condition throughout the gait cycle, (2) there were no significant differences across brain regions between the sSC and SC conditions within the O and T groups throughout the gait cycle, and (3) a significant difference was found in the sSC conditions between the O and T groups. Specifically, there was a significant difference in AmEEGDel at the central frontal position during the loading response phase and the frontocentral position during the mid stance phase of the gait cycle. These findings may have implications for rehabilitation practitioners, especially when tailoring interventions for patients undergoing gait rehabilitation on different walking surfaces.

### 4.1. Between-Group Differences in EEG Activity across the Gait Cycle

In our study, we observed similar brain activity in the SC condition between the O and T groups across various brain frequency bands during the gait cycle. Previous research on the impact of auditory cueing on cortical activity during walking on different surfaces is limited. However, contrary to our observations, Roeder et al. [23] reported a significant increase in the magnitude of cortical activity in the theta frequency band during the double-support (mid stance) phase when walking overground compared to on a treadmill at participants’ preferred speed (0.92–1.33 m/s or 3.3–4.8 km/h). Without the influence of an auditory cue, Roeder et al.’s [23] findings align with the expectation that differences in kinematics, kinetics and electromyographic (EMG) activity between these two surfaces [37] independently modulate brain activity. In our study, it is challenging to explain the lack of a marked difference in cortical activity in the SC condition between overground (auditory cue) and treadmill walking (non-auditory cue). However, it may be speculated that the task of stepping overground in synchronization with the auditory cue at the speed used in our study (0.55 m/s, 2.88 km/h) provided too little mental engagement to lead to a noticeable increase in cortical activity. Alternatively, increased cortical activity may have been present with the auditory cue, but potentially masked by participants also having increased cortical activity when walking on a treadmill.

In contrast to the similar brain activity observed in the SC condition between groups, we found a marked difference in brain activity in the sSC condition. Specifically, the central frontal brain area in the delta frequency band exhibited a significantly increased activity in TsSC compared with OsSC during the loading response phase (3.7–7.2%; Figure 3a) of the gait cycle. Moreover, the frontocentral position in the delta frequency band was significantly increased in TsSC compared with OsSC at the mid stance phase (28.2–30.8%; Figure 3b) of the gait cycle. It is understood that the frontocentral area plays an important role in brain processing during walking intention, particularly in gait initiation and the generation of movement-related cortical potentials during stepping [38]. This has previously been demonstrated during a walking task using a brain–computer interface (BCI) requiring participants to control an avatar’s walking [39]. The central frontal cortex is interconnected with cortical motor areas in humans [40] and plays a pivotal role in movement-related motor cortex and visuomotor processing [41]. Taken together, the increased brain activation in both the frontocentral and central frontal cortex in the delta frequency band suggests that treadmill walking results in a greater cognitive load compared with overground walking at similar self-selected speeds. In TsSC, brain function in the frontal area may require precise motor planning and timing adjustments to synchronize with the belt’s motion. It has previously been proposed that walking at a fixed speed on a treadmill is monotonous and requires little mental engagement for motor learning [25]. However, our current data suggest that treadmill walking actually requires greater mental engagement than overground walking (at similar speed) during the gait phase. Nevertheless, this finding may simply be a result of participant unfamiliarity with walking on a treadmill.

### 4.2. Within-Group Differences in EEG Activity across the Gait Cycle

Another key finding from our study indicated consistent EEG activity across various brain regions during the gait cycle, irrespective of whether participants were in the sSC or SC condition. In the O group, our results suggest that the introduction of an auditory cue during overground walking does not alter brain activity across the gait cycle compared to walking at a similar self-selected walking speed (for the same duration). This finding contradicts previous research suggesting heightened brain activity when processing auditory and sensorimotor-related information [28,42]. For instance, past studies have linked the left temporal cortex to responses to auditory cues, such as synchronizing finger-tapping with a metronome [43]. This brain area typically plays a role in receiving and interpreting auditory cues related to a desired walking speed. Earlier investigations also noted changes in amplitude in the parieto-occipital region during overground walking, particularly when participants simultaneously engaged in visual discrimination tasks using virtual reality [44] or precision stepping [28]. However, our findings do not align with the idea of increased utilization of visual and sensory processing resources in the central parieto-occipital area, which is conventionally thought to be actively involved in processing visual information and responding to external stimuli [45]. Given the absence of differences between the OsSC and OSC conditions, our results challenge the notion that the convergence of visual, tactile and auditory information occurs in the parietal regions with auditory cueing during overground walking. This convergence is commonly recognized as a key sensorimotor interface facilitating multisensory integration during ongoing movements [46,47]. The discrepancy between our findings and previous research questions the extent to which auditory cues significantly impact brain activity during overground walking.

Similarly, we found no significant difference in brain activity across the gait cycle between the sSC and SC conditions in the T group. In the SC condition, participants walked on the treadmill at a fixed speed (0.55 m/s, 2.88 km/h), which matched the speed of overground walking accompanied by an auditory cue. This allowed for a direct comparison between groups in the SC condition. In contrast, in the sSC condition, participants walked at a self-selected speed not exceeding 0.8 m/s (2.88 km/h). Despite the same walking duration (3 min) and brain activity in both the TSC and TsSC conditions (and in the O group), a discrepancy in step frequency was noted (Table 1). This could potentially complicate the interpretation of brain activity derived from EEG time-series data when making comparisons between conditions. Although this may be considered a study limitation, we argue that permitting participants to select their walking speed offers a more ecologically valid comparison, especially considering the limited alternatives for controlling walking speed without the use of auditory or visual cues.

### 4.3. Methodological Considerations

In this study, a significant methodological consideration concerns the potential influence of movement artifacts in the EEG recording. To mitigate this effect, we controlled walking speed at 0.55 m/s (1.98 km/h) in the SC condition, consistent with previous studies [29,30]. Additionally, participants were instructed not to exceed a walking speed of 0.8 m/s (2.88 km/h) in the sSC condition, as previous research has indicated that movement artifacts become more pronounced beyond this threshold [28]. Furthermore, ICA was employed to remove artifacts from EEG data obtained during movement. ICA is a well-established and widely utilized artifact removal method for EEG data during walking and movement tasks [20,21,33,34,48,49,50,51,52,53,54,55]. The fast and highly efficient fixed-point fastICA algorithm was specifically utilized in this study to separate original signals from artifacts present in contaminated EEG signals. The FastICA algorithm effectively addresses ocular originated artifacts and glossokinetic artifacts, respectively [33,34,52]. However, it is important to acknowledge that ICA typically requires a large amount of data for accurate component estimation. Consequently, the optimization process can be computationally intensive and time-consuming. This limitation warrants consideration, particularly in the context of clinical applications and the utilization of BCI.

Several methodological considerations could potentially offer additional insights into our data. While this study employed slow walking speeds (0.55 m/s and <0.8 m/s) to minimize movement artifacts in EEG recordings, it is acknowledged that these walking speeds may not fully represent muscle activity or natural gait speeds. Future studies should explore cortical activity during more natural gait speeds, employing EEG recordings less susceptible to movement artifacts and utilizing more sophisticated artifact removal techniques.

## 5. Conclusions

Our EEG data suggest a minimal impact of employing an auditory cue during overground walking to increase cortical processing, as we did not observe an increase in brain activity compared to walking without an auditory cue. Furthermore, treadmill walking at a self-selected speed appears to elicit heightened brain activity compared with walking overground at a similar speed, suggesting that treadmill walking requires enhanced motor coordination and synchronization under these conditions. These findings provide insights with potential implications ranging from walking rehabilitation and the development of volitional control systems for neuroprostheses to enhancing complex movements in sports performance.

## Figures and Tables

**Table 1:** The number of steps in 3 min (stride cycle) in each walking condition.

Subject ID	Group	Speed Condition		Subject ID	Group	Speed Condition	
		sSC	SC			sSC	SC
N1	T	105	68	N13	O	72	69
N2	T	119	68	N14	O	93	68
N3	T	129	69	N15	O	89	67
N4	T	113	68	N16	O	106	69
N5	T	76	68	N17	O	91	69
N6	T	103	68	N18	O	81	69
N7	T	110	67	N19	O	99	68
N8	T	124	69	N20	O	84	67
N9	T	134	67	N21	O	79	67
N10	T	119	69	N22	O	65	68
N11	T	137	68	N23	O	78	68
N12	T	131	67	N24	O	124	68
Average steps (SD)	T	116.7 (17.0)	68 (0.7)	Average steps	O	88.4 (16.0)	68 (0.8)

T: treadmill walking, O: overground walking; sSC: walking with self-selected speed control, SC: walking with speed control.

## Data Availability

The data that support the findings of this study are available at https://osf.io/2R9BA/ (accessed on 9 January 2024).
